# Comparison of MRI Sequences to Predict *IDH* Mutation Status in Gliomas Using Radiomics-Based Machine Learning

**DOI:** 10.3390/biomedicines12040725

**Published:** 2024-03-25

**Authors:** Dilek N. G. Kasap, Nabila Gala Nacul Mora, David A. Blömer, Burak Han Akkurt, Walter Leonhard Heindel, Manoj Mannil, Manfred Musigmann

**Affiliations:** University Clinic for Radiology, University of Münster, University Hospital Münster, Albert-Schweitzer-Campus 1, DE-48149 Münster, Germany; dilek.kasap@uni-muenster.de (D.N.G.K.); nabilagala.naculmora@ukmuenster.de (N.G.N.M.); musigma@uni-muenster.de (M.M.)

**Keywords:** glioma, *IDH* mutation status, machine learning, artificial intelligence, neuroimaging, MRI, radiomics

## Abstract

Objectives: Regarding the 2021 World Health Organization (WHO) classification of central nervous system (CNS) tumors, the isocitrate dehydrogenase (*IDH*) mutation status is one of the most important factors for CNS tumor classification. The aim of our study is to analyze which of the commonly used magnetic resonance imaging (MRI) sequences is best suited to obtain this information non-invasively using radiomics-based machine learning models. We developed machine learning models based on different MRI sequences and determined which of the MRI sequences analyzed yields the highest discriminatory power in predicting the *IDH* mutation status. Material and Methods: In our retrospective IRB-approved study, we used the MRI images of 106 patients with histologically confirmed gliomas. The MRI images were acquired using the T1 sequence with and without administration of a contrast agent, the T2 sequence, and the Fluid-Attenuated Inversion Recovery (FLAIR) sequence. To objectively compare performance in predicting the *IDH* mutation status as a function of the MRI sequence used, we included only patients in our study cohort for whom MRI images of all four sequences were available. Seventy-one of the patients had an *IDH* mutation, and the remaining 35 patients did not have an *IDH* mutation (*IDH* wild-type). For each of the four MRI sequences used, 107 radiomic features were extracted from the corresponding MRI images by hand-delineated regions of interest. Data partitioning into training data and independent test data was repeated 100 times to avoid random effects associated with the data partitioning. Feature preselection and subsequent model development were performed using Random Forest, Lasso regression, LDA, and Naïve Bayes. The performance of all models was determined with independent test data. Results: Among the different approaches we examined, the T1-weighted contrast-enhanced sequence was found to be the most suitable for predicting *IDH* mutations status using radiomics-based machine learning models. Using contrast-enhanced T1-weighted MRI images, our seven-feature model developed with Lasso regression achieved a mean area under the curve (AUC) of 0.846, a mean accuracy of 0.792, a mean sensitivity of 0.847, and a mean specificity of 0.681. The administration of contrast agents resulted in a significant increase in the achieved discriminatory power. Conclusions: Our analyses show that for the prediction of the *IDH* mutation status using radiomics-based machine learning models, among the MRI images acquired with the commonly used MRI sequences, the contrast-enhanced T1-weighted images are the most suitable.

## 1. Introduction

Gliomas, a diverse group of central nervous system (CNS) tumors, present an intricate challenge in the realm of neuro-oncology due to their diverse clinical behaviors and complex molecular underpinnings [1]. Accurate characterization and classification of gliomas are crucial for guiding treatment decisions and predicting patient outcomes [2]. With the release of the 2021 World Health Organization (WHO) classification guidelines [3], the landscape of glioma classification underwent a profound transformation [4,5,6]. Central to this classification are molecular markers, notably the isocitrate dehydrogenase (*IDH*) and alpha thalassemia/mental retardation syndrome X-linked (*ATRX*) mutations, which have emerged as pivotal elements in characterizing gliomas and informing clinical decision making [1,7,8].

*IDH*-mutant gliomas tend to initially present as lower histologic grades with better outlooks, boasting a median survival of over 12 years [9]. However, they frequently transform to higher grades as the disease progresses. On the other hand, *IDH* wild-type gliomas typically manifest as glioblastomas (GBMs), which are the most prevalent and highly aggressive grade IV brain tumors according to the WHO classification [3]. Despite aggressive treatments involving multiple approaches, the average survival for individuals with *IDH* wild-type gliomas is only 12 to 15 months [10,11].

Additionally, the presence or absence of *ATRX* mutations and of the *1p/19q* co-deletion status further refines our ability to dissect glioma heterogeneity [12]. The *ATRX* gene holds significant relevance in various types of tumors, particularly adult-type diffuse glioma [13]. This gene is pivotal in the process of chromatin remodeling and the preservation of genomic stability [14].

As the diagnostic landscape of gliomas evolves, non-invasive methods for assessing molecular markers are becoming increasingly important. Magnetic resonance imaging (MRI) is the preferred method in the diagnosis and characterization of gliomas, offering valuable insights into their structure and function [15,16]. Specific MRI sequences such as T1-weighted native (T1w native), T1-weighted with contrast enhancement (T1w CE), T2-weighted (T2w), and Fluid-Attenuated Inversion Recovery (FLAIR) are commonly employed for this purpose [17,18]. It is worth noting that magnetic resonance spectroscopy has emerged as a promising tool for predicting the *IDH* mutation status in gliomas, contributing to the advancing diagnostic precision in glioma classification. Suh et al. and Bauer et al. investigated the role of 2-Hydroxyglutarate as a predictor of the *IDH* mutation in gliomas with very promising results [19,20]. In recent years, radiomics, a cutting-edge field at the intersection of medical imaging and data science, has emerged as a promising approach to extract valuable information from medical images, including magnetic resonance imaging, and transform it into clinically meaningful insights [21,22]. Radiomics, at its core, harnesses the information embedded within medical images, by extracting a multitude of quantitative features [21,22]. These radiomic features encompass a wide array of data, including textural, morphological, and functional characteristics, which have the potential to offer unique insights into glioma subtypes and their underlying molecular alterations [23,24]. The combination of radiomics based on MRI and machine learning offers numerous potential applications in neuroradiological diagnostics. For example, Alves et al. investigated the differentiability of inflammatory lesions and brain tumors using such methods [25]. Radiomics, when coupled with machine learning techniques, has also demonstrated its potential in predicting various molecular markers and clinical outcomes in diverse malignancies [26,27,28]. In this context, machine learning refers to the use or development of algorithms that are able to independently recognize patterns in data and subsequently draw their own conclusions based on the previously learned relationships. In glioma research, radiomics-based machine learning models have shown promise in non-invasively predicting molecular alterations using MRI data [29,30]. By integrating radiomics analysis with molecular information, there is an opportunity to refine and enhance the precision of glioma classification.

In alignment with our study, current research in the field of neuroimaging has investigated the efficacy of radiomics models in non-invasively predicting mutation status in gliomas [26,31,32,33,34,35]. Choi et al. employed a fully automated segmentation model based on Convolutional Neural Networks (CNNs) for multi-parametric MRI images, demonstrating its effectiveness in predicting the *IDH* mutation status [31]. W.Y. Huang et al., using radiomics analyses, found the radiomics model based on T1w CE to perform better than models based on other sequences in predicting tumor grade and *IDH1* status of gliomas [34]. Additionally, L. Han et al. conducted an MRI texture analysis, emphasizing the suitability of the T1w CE sequence for predicting *IDH1* mutations [35]. Conversely, some studies suggest the T2w sequence to be the most suitable for predicting the *IDH* mutation status [26,33].

We are interested in predicting the *IDH* mutation status, i.e., distinguishing between *IDH*-mutated and *IDH* wild-type gliomas using radiomics-based machine learning. However, the specific aim of our current study is not to predict *IDH* mutation status with the highest possible accuracy that can somehow be achieved using machine learning models. Rather, we are interested to determine which of the commonly used MRI sequences is best suited for this diagnostic task. For this purpose, we compared the results obtained with different machine learning algorithms using native and contrast-enhanced T1-weighted MRI images, T2-weighted MRI images, and MRI images acquired with the FLAIR sequence.

## 2. Materials and Methods

Our study was performed in compliance with the Declaration of Helsinki and approved by the local ethics committee (Ärztekammer Westfalen-Lippe, ÄKWL 2021-596-f-S). Due to its retrospective nature, written informed consent was waived. We aimed to analyze which of the commonly used MRI sequences is best suited to determine *IDH* mutation status non-invasively using radiomics-based machine learning models. As already explained, *IDH* mutation status is one of the most important markers for the classification of diffuse gliomas in adults according to the 2021 WHO classification of CNS tumors. We searched our database for patients diagnosed with glioma between October 2007 and December 2021. In our study cohort, only patients with (1) native, T1-weighted; (2) contrast-enhanced (CE), T1-weighted; (3) T2-weighted; and (4) FLAIR sequence MRI images were included. We only included patients for whom MRI images of all four sequences mentioned were available to compare the performance achievable with the different sequences in terms of predictability of *IDH* mutation status as objectively as possible. The MRI sequences utilized in our study were obtained from various hospitals and radiology practices, resulting in a combination of images captured using both 1.5 Tesla and 3 Tesla MRI machines. Despite the variability in magnetic field strength, we ensured uniformity across all included patients by employing consistent imaging protocols. This standardization of imaging protocols allowed for a comprehensive analysis of radiomic features across different MRI sequences, irrespective of the magnetic field strength utilized during the imaging process.

Initially, 107 patients were included in our study. We excluded one of these 107 patients due to unknown *IDH* mutation status. No further patients were excluded. The final cohort of 106 patients contained 71 patients with an *IDH* mutation and 35 patients without an *IDH* mutation (*IDH* wild-type). The demographic characteristics of the data used to compare the MRI sequences in terms of predictability of *IDH* mutation status are summarized in Table 1.

### 2.1. Radiomics

Segmentation of the entirety of each tumor was semi-automatically performed by two radiology residents in their 3rd year of residency and two medical students with experience in MRI segmentation. Additionally, all images were visually assessed by a board-certified neuroradiologist with 11 years of experience. We utilized the 3D slicer open-source software platform (version 4.10, October 2018, www.slicer.org, accessed on 1 December 2021) and the Segmentation Wizard plugin. Expert consensus from two radiologists with five and ten years of experience in neuroimaging was used in anatomically difficult segmentations. 

For each of the four MRI sequences used, we determined a total of 107 radiomic features using the PyRadiomisc package. The package is available as an implementable plugin for the 3D slicer platform. The 107 radiomic features were extracted by hand-delineated regions of interest (ROI) from the MRI images of each patient. They were assigned to the seven different feature classes: “first-order statistics” (18 features), “shape-based features” (14 features), “gray-level co-occurrence matrix features” (24 features), “gray-level run-length matrix features” (16 features), “gray-level size-zone matrix features” (16 features), “neighboring gray-tone difference matrix features” (5 features), and “gray-level dependence matrix features” (14 features). The numerical values in the brackets indicate the respective number of features belonging to the corresponding feature class. The longlist used for model development initially contained all 107 radiomic features, the age of the patients at the time of diagnosis, and their gender. Redundant features were subsequently removed from the longlist using a 95% correlation filter. All features were standardized.

### 2.2. Statistical Analysis

We performed our statistical analysis using R software (version 4.1.2, November 2021). The 106 patients included in our final study cohort were assigned randomly to a training group and an independent test group, using a stratified 4:1 ratio with a balanced distribution of *IDH*-mutated and *IDH* non-mutated patients between these two groups (see Table 1). We assigned the data/MRI images generated with the four sequences T1w native, CE, T2w, and FLAIR in each case identically to the respective training groups or independent test groups to be able to compare the results obtained with these four different sequences as objectively as possible. A total of 80% of the total data (i.e., the training data) were used for the feature preselection and subsequent model development. We performed feature preselection and model development using four different machine learning algorithms: Random Forest, Lasso (least absolute shrinkage and selection operator) regression, linear discriminant analysis (LDA), and Naïve Bayes. We used 10-fold cross-validations to optimize the hyperparameters included in the models. The models themselves were optimized by maximizing the area under the curve (AUC) of the receiver operator characteristic (ROC). Finally, the achieved model performance was tested using the independent test data (i.e., using the remaining 20% of the total data). The discriminatory power of our models was determined in terms of AUC, accuracy, sensitivity, specificity, positive predictive value (PPV), and negative predictive value (NPV). With respect to our analyses, sensitivity describes the proportion of correctly predicted cases with an *IDH* mutation. Accordingly, specificity describes the fraction of correctly predicted cases without an *IDH* mutation (*IDH* wild-type). The positive predictive value indicates the proportion of correctly predicted cases with an *IDH* mutation relative to all predicted cases with an *IDH* mutation. Finally, the negative predictive value describes the rate of correct predictions of cases without an *IDH* mutation (*IDH* wild-type) relative to all predictions of cases without *IDH* mutation.

Because the performance achievable with the models depends on the number of features included in the models and to rule out possible overfitting, we started by constructing each model multiple times with an increasing number of features. We always started with univariate models. Only the most important features were included in the models. These were previously determined based on the training data using the “varImp” function in R (varImp = variable importance). The varImp function determines the significance of the individual features by determining the model performance with and without a specific feature. The significance of the individual features is subsequently determined based on a comparison of the respective performance results. We identified the optimal and final number of features to include in the models by determining which number of features achieved the highest model performance in relation to the independent test data. Our approach minimized the risk of possible overfitting.

**Figure 1:**
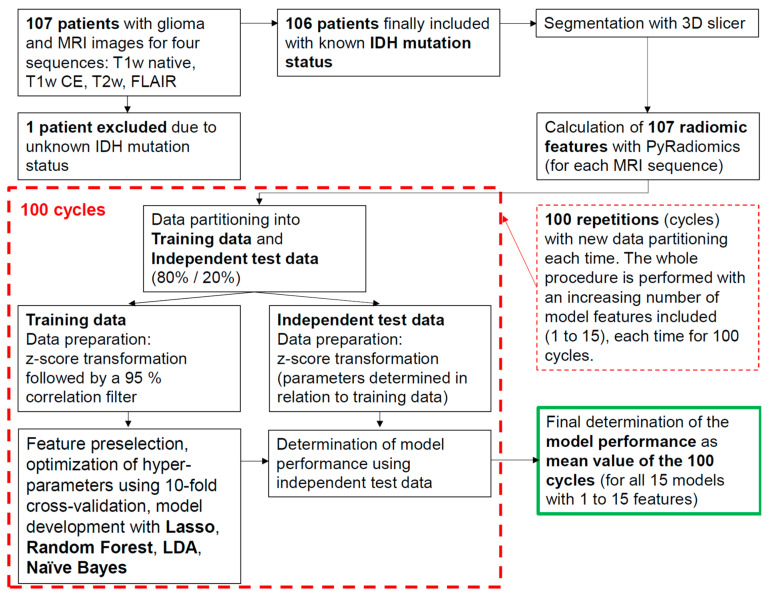
Flowchart describing the methodological approach. Four machine learning algorithms to predict *IDH* mutation status were tested: Lasso regression, Random Forest, LDA, and Naive Bayes. All models were developed with an increasing number (1 to 15) of features and for each of the four MRI sequences analyzed (T1 native, T1 CE, T2, and FLAIR). Each of these models was developed 100 times with new data partitioning each time and tested with independent test data afterward. The final model performances were determined as the average values of the 100 runs in each case.

Both the exact model compositions and the discriminatory power obtained depended slightly on the specific division of the data into training and independent test data used. Our aim was to eliminate such slight random effects associated with data partitioning as much as possible and thus to demonstrate the robustness of our approach and its results. We therefore performed the data partitioning and subsequent model development a total of 100 times, each time using a new partitioning of the data into training and independent test data. All performance values were calculated based on the associated independent test datasets and as averages of these 100 cycles. A detailed description of the methodology we used can be found in Musigmann et al. [36]. For better understanding, we have additionally described the entire methodological approach in a flowchart (see Figure 1). 

It should be noted that the described procedure with 100 repetitions can, in principle, lead to up to 100 slightly different models. Therefore, based on the model variant that yielded the highest discriminatory power overall, we additionally created two final models that contained, in a fixed manner, only the features that were most frequently selected in the associated 100 runs previously conducted. The first of these two models contained 10 features. This is the number of features belonging to the model that exhibited the highest discriminatory power of all our tested model variants. In the second model, only the features that were selected in at least 50% of the previously performed 100 runs were considered. This applied to the seven most important features. The procedure used to develop these two models with 7 and 10 included features, respectively, was the same as the approach described previously. This means that again 100 different data partitions were used, and all discriminatory power values were calculated as mean values of the 100 repetitions and with respect to the respective independent test data.

## 3. Results

### 3.1. Results of Comparing MRI Sequences to Predict IDH Mutation Status 

**Figure 2:**
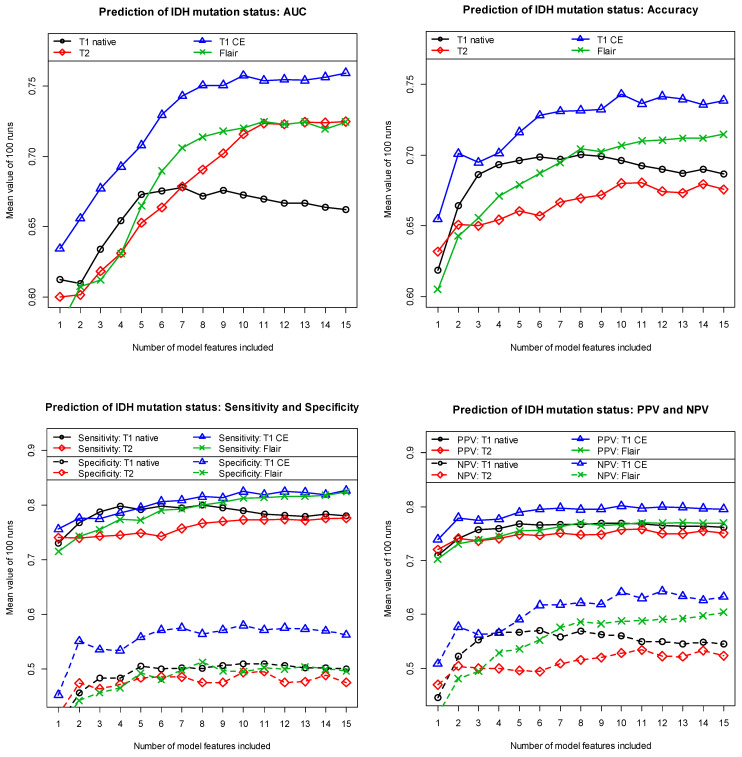
Mean discriminatory power (using 100 runs) in the prediction of *IDH* mutation status in gliomas using Lasso regression based on MRI images of four different sequences. AUC = area under the curve; PPV and NPV = positive and negative predictive values. All results were calculated using independent test data.

As explained earlier, we tested four machine learning algorithms to predict *IDH* mutation status: Random Forest, Lasso regression, LDA, and Naive Bayes. Each of these four algorithms was performed with an increasing number of features included in the models. At this point, however, it should be pointed out once again that the specific aim of this study was not to predict *IDH* mutation status with the highest possible accuracy that can somehow be achieved using radiomics-based machine learning models. The aim of this study was to determine which of the commonly used MRI sequences is best suited for this purpose. We used MRI images acquired with the T1 sequence (with and without contrast administration), the T2 sequence, and the FLAIR sequence. Based on the four machine learning algorithms we tested (each algorithm was tested with MRI images acquired with each of the four different MRI sequences), we obtained the best results using Lasso regression. In comparison, we received slightly worse results with the Random Forest algorithm. The worst results were obtained using LDA and the Naive Bayes algorithm. Figure 2 shows the discriminatory power obtained with our models using Lasso regression in terms of AUC (top left), accuracy (top right), sensitivity and specificity (bottom left), and, finally, positive and negative predictive values (bottom right) as a function of the number of features included in the models. The figures show the average values determined with the respective independent test data. All results were calculated based on 100 different data partitions of the data into training and independent test data. Comparing the results obtained with the four different MRI sequences, it is evident that *IDH* mutation status can be most accurately predicted using T1-weighted contrast-enhanced MRI images. The T2-weighted and the native T1-weighted MRI images are found to be the worst for this task. Finally, the results obtained with the MRI images generated using the FLAIR sequence are in the middle range.

In the case of the three sequences T1w CE, T2w, and Flair, the inclusion of additional features in the model leads to an increase in discriminatory power approximately up to the ninth feature. When using the native T1-weighted MRI images, the discriminatory power increases only up to the sixth feature. If more than nine (six) features are used, the discriminatory power no longer increases significantly or even decreases again. This indicates the beginning of an overfitting. However, regardless of the number of final features included in the models, T1-weighted contrast-enhanced MRI images almost always yielded the highest discriminatory power in predicting the *IDH* mutation status.

At this point, one might ask whether a different machine learning approach other than the Lasso regression we used would not have resulted in a different MRI sequence other than the T1-CE sequence we found. Three remarks are worth mentioning in this context:

(1) As described, we tested not only Lasso regression but also Random Forest, LDA, and Naive Bayes for predicting the *IDH* mutation status. With all four machine learning algorithms, the contrast-enhanced T1-weighted MRI images were invariably and unequivocally found to be the most suitable for predicting the *IDH* mutation status. The images generated using the FLAIR sequence produced the second-best results on average. Accordingly, using the native T1-weighted or the T2-weighted MRI images yielded the worst results on average.
(2) In addition, we also calculated and compared the univariate discriminatory power for all features included in the longlist for each of the four MRI sequences tested. These values do not depend on the machine learning algorithm subsequently used. Here, several features derived from the contrast-enhanced T1-weighted MRI images also proved to be the most discriminative factors. Some of the features derived based on the FLAIR sequence also showed promising univariate discriminatory power. In contrast, the features derived from the native T1-weighted MRI images showed the lowest discriminatory power on average, which is again consistent with Figure 2. Of course, these results do not mandatorily apply to a multivariate approach as well, but they can be interpreted as an additional indication that contrast-enhanced T1-weighted images are likely to provide the best results with respect to our diagnostic question.
(3) Finally, we also developed models using Lasso regression, where we simultaneously provided the features of all four MRI sequences for feature selection. Similar to the results of our univariate analyses, features determined with contrast-enhanced T1-weighted MRI images and with the FLAIR sequence again proved to be particularly important. However, the models developed using the features of all four sequences simultaneously did not show a higher discriminatory power on average than the models based solely on the contrast-enhanced T1-weighted MRI images.

We would like to briefly point out here that our analyses not only determined the most appropriate MRI sequence for predicting the *IDH* mutation status but also identified the added value of contrast administration with respect to this diagnostic question. The added value obtained using a contrast agent can be calculated directly using Figure 2 as the difference of the curves determined for the T1-weighted native and contrast-enhanced MRI images. It is obvious that the administration of the contrast agent significantly increased the discriminatory power.

### 3.2. Results of Comparing MRI Sequences to Predict IDH Mutation Status Using Fixed Features

As explained earlier, due to the 100 different partitions into training and independent test data, followed by new feature selection and model development in each cycle, the final 100 models may in principle be composed differently. The question is how different the final models really are in terms of their specific feature composition. Therefore, for each feature that was included in at least one of the 100 models developed with Lasso regression and the contrast-enhanced T1-weighted MRI images, we determined the number of 100 runs in which the feature was selected. Table 2 lists the frequencies of the most frequently selected features. The numbers indicate the respective frequency of selection of the associated feature during the 100 cycles. The results were calculated for the 10-feature models.

It turns out that the 100 models are very similar in their composition of features. The two features “age_at_diagnosis” and “original_shape_Flatness” are almost always included. Two further features were selected in more than three out of four runs (in 86% and 78% of all runs, respectively). Three additional features were included in at least more than every second model (in 61%, 55%, and 52% of all runs). All other features listed in Table 2 were already selected in less than 50% of all runs and quickly lost importance. This fact is consistent with the observation that for the T1-CE sequence, discriminatory power increases only slightly from about the seventh feature onward and not at all from the tenth feature onward (see Figure 2).

We were interested in the extent to which the performance achieved with our models depended on the exact composition of the individual models. Therefore, we developed two additional models with fixed features. The first model contains only those features which were selected in at least 50% of all runs, i.e., the first seven features listed in Table 2. The second model includes the 10 most important features in a fixed manner (i.e., “age_at_diagnosis” to “original_glszm_ZonePercentage”, see Table 2). Based on the independent test data and the contrast-enhanced T1-weighted MRI images, we received the highest discriminative power with our 10-feature models. These two models were again developed 100 times with different data partitions, and the performance was subsequently determined using the independent test data. This time, however, the features included were fixed within the 100 runs.

The classification results for predicting *IDH* mutation of these 7- and 10-feature models are summarized in Table 3. According to the method used to calculate the results shown in Figure 2, the columns labeled “Different features” contain the results for the models with newly determined relevant features in each of the 100 runs. The columns labeled “Fixed features”, on the other hand, contain the results for the models calculated with the fixed features according to Table 2. All results were again calculated with independent test data and as mean values of 100 runs. The numbers in brackets in the table indicate the 95% confidence intervals of the results. Comparing the 7-feature and the 10-feature models, it is evident that the additional three features yield only a very moderate increase in discriminatory power (see also Figure 2). The models show high performance in predicting the *IDH* mutation status. The 7-feature models yield the following average performance values (different features/fixed features): mean AUC = 74.3%/84.6%, mean accuracy = 73.1%/79.2%, mean sensitivity = 80.9%/84.7%, mean specificity = 57.6%/68.1%, mean PPV = 79.8%/84.8%, and mean NPV = 61.8%/71.0%. Our described methodology with 100 (or even more) runs is well suited to compare different approaches and to investigate the stability of the respective model approaches. The models with the fixed features show a slightly higher discriminatory power compared to the models with different features. The comparison of the widths of the individual confidence intervals indicates that the approach with the fixed features also leads to more stable results. Overall, however, both approaches (with different and fixed features) yield high discriminatory power in predicting the *IDH* mutation status.

## 4. Discussion

*IDH* mutation status has gained paramount importance in glioma classification since the 2021 WHO guidelines were released. It is not just a marker but a key determinant of clinical behavior and patient outcome. The accuracy of identifying *IDH* mutations non-invasively through MRI sequences holds immense clinical value. 

While biopsy remains the gold standard for molecular diagnosis, it is often hindered by associated risks, including mortality, seizure, impaired consciousness, brain swelling, and hemorrhaging [37]. Moreover, in certain clinical scenarios, conducting a comprehensive MRI examination with all recommended sequences may not be feasible. Therefore, our study aimed to provide a non-invasive alternative for *IDH* mutation prediction, particularly catering to situations where a biopsy is impractical or MRI sequences are limited. The results we obtained shed light on the most effective MRI sequence for this crucial diagnostic task and underscore the critical role of contrast-enhanced T1-weighted MRI images. 

Based on the results of our study, we specifically propose the use of contrast-enhanced T1-weighted MRI images for the non-invasive prediction of the *IDH* mutation status using machine learning algorithms.

Our patient cohort consisted of 106 individuals, comprising 71 with *IDH* mutations and 35 with *IDH* wild-type gliomas. We employed four common MRI sequences to extract radiomic features encompassing various data classes. To ensure the robustness of our findings, we randomly divided the patients into training and independent test groups through 100 iterations, maintaining a balance between *IDH*-mutated and wild-type cases. We leveraged four machine learning algorithms including Lasso regression, Random Forest, LDA, and Naïve Bayes for feature preselection and model development. Hyperparameters were optimized via 10-fold cross-validation. Model performance was rigorously assessed using independent test data. To mitigate overfitting, we determined the optimal number of features for each model and identified feature importance through the varImp function. In our study, we placed great emphasis on avoiding possible overfitting. Therefore, we developed each model 100 times using different training datasets and determined the average performance values during these 100 runs based on independent test data. We also reduced the number of features included in the final models as much as possible and analyzed how often these features were selected during the 100 runs (i.e., we ended up using only the best-ranked features). The importance of avoiding overfitting in machine learning is also discussed in more detail by Dietterich [38], for example.

In our comprehensive analysis, which involved various machine learning algorithms, Lasso regression consistently emerged as the superior model for predicting the MRI sequence, highlighting its unique diagnostic value for predicting the *IDH* mutation status. Among the tested MRI sequences in our study, contrast-enhanced T1-weighted images consistently demonstrated the highest suitability for predicting the *IDH* mutation status. This sequence consistently exhibited superior performance metrics, including an average AUC of 0.846, an average accuracy of 0.792, an average sensitivity of 0.847, and an average specificity of 0.681, as determined by the seven-feature model developed with Lasso regression. Our analysis also explored the impact of feature selection and model complexity. It was observed that increasing the number of features in the models up to 10 features led to improved performance, after which overfitting became a concern. Additionally, it was found that the administration of contrast agents significantly contributed to the enhancement of discriminatory power, highlighting the significance of this approach. Interestingly, human readers were unable to assess *IDH* mutation status by mere visually assessment in a subset of 20 patients with contrast-enhancing gliomas (sensitivity 100% (95% CI 69.15% to 100.00%) and specificity 0% (95% CI 0.00% to 30.85%)).

Our study also revealed that the FLAIR sequence is moderately suitable for this purpose, while the T1w and T2w native sequences were identified as the least suitable options. Comparing our findings to those in the existing literature (as highlighted in the introduction), it is noteworthy that there has been limited exploration on the identification of the most effective MRI sequence for the non-invasive prediction of the *IDH* mutation status in gliomas. Our study contributes significantly to this gap by demonstrating the superiority of contrast-enhanced T1w MRI images for this purpose.

The varied findings in the current literature underscore the complexity of the relationship between MRI sequences and the prediction of the *IDH* mutation status. The discrepancies across studies could be attributed to several factors. Firstly, the unique microenvironment of gliomas may influence the performance of different MRI sequences. Gliomas exhibit considerable heterogeneity in terms of vascularity and cellularity and the presence of necrotic regions, presenting challenges in accurately capturing these diverse characteristics through imaging modalities. It is possible that specific characteristics of our patient cohort favored the discriminative power of contrast-enhanced T1w images.

Secondly, the choice of imaging modality may be influenced by the radiomic features extracted and the machine learning algorithms employed. Recent advancements in radiomics have introduced various approaches to feature extraction, and the selection of optimal features may vary across studies. Additionally, the choice of machine learning algorithms can significantly impact the performance of predictive models. In our study, the consistent superiority of contrast-enhanced T1w images may be attributed to a combination of feature selection, algorithm choice, and specific radiomic characteristics captured by T1w images.

Despite this diversity, our study contributes to the ongoing discourse on the optimal choice of MRI sequences in glioma research and adds valuable insights into the evolving field of radiomics. As our study aligns with the therapeutic guidelines set by the German Association of Neurology and Neurosurgery, emphasizing the importance of the WHO’s classification in determining the standard of care for gliomas, it further supports the integration of non-invasive techniques into routine clinical practice.

Regarding model stability, our study demonstrated that the selected features were robust across multiple runs, suggesting that our findings are reliable and not overly influenced by random effects associated with data partitioning.

As already mentioned in the introduction, the primary aim of our current study was neither to achieve the highest possible discriminatory power in the non-invasive prediction of the *IDH* mutation status using a conventional machine learning algorithm or a deep learning approach, nor to find the most suitable algorithm for this task. Rather, the aim of our study was to find out which of the commonly used MRI sequences is best suited for this important diagnostic task. New, promising medical image segmentation methods could lead to even more precise results for diagnostic questions such as the one considered in this study. Interesting ideas in this regard are semi-supervised medical image segmentation (see Zhao et al. [39]) and transformer-based approaches [40,41].

The molecular and histological characteristics of diffuse gliomas of the adult type are decisive for their classification, treatment strategies, and prognosis. The current standard treatment of gliomas is based on adhering to the guidelines established by the German Association of Neurology and Neurosurgery according to their WHO classification. This therapeutic approach typically involves a multimodal strategy encompassing surgical intervention or tumor reduction; chemotherapy with either procarbazine/lomustine/vincristine (PCV) or temozolomide (TMZ); and radiotherapy [42].

Of particular significance is the role of the *IDH* mutation status in gliomas. Beyond aiding in the initial classification of gliomas, the *IDH* mutation status holds predictive value for treatment responses. Notably, *IDH* mutations have been linked to heightened sensitivity to certain chemotherapeutic agents, such as Temozolomide [43]. This heightened sensitivity can translate into improved response rates and longer progression-free intervals in patients with *IDH*-mutated gliomas. This finding further underscores the critical importance of being able to predict *IDH* mutation status in gliomas.

It is worth noting that in our previous study by Nacul Mora et al. [44], we were able to successfully predict the *ATRX* status in gliomas using radiomics-based machine learning. Notably, T2-weighted MRI sequences emerged as being the most effective in predicting *ATRX* status in gliomas, indicating their pivotal role in molecular classification. This finding aligns with our observations regarding the potential of specific MRI sequences in providing valuable insights into the molecular landscape of gliomas.

Despite the valuable insights gained from our study, several limitations should be acknowledged. Firstly, due to the retrospective nature of our research, some patient data were incomplete, leading to the exclusion of 1 of the 107 patients. Additionally, while our study represents a substantial contribution to the field, further validation in larger cohorts and exploration of avenues for clinical translation are warranted to fully harness the clinical implications of these findings. An interesting way to further validate our results could also be the use of external databases such as the Rembrandt database (REpository for Molecular BRAin Neoplasia DaTa) of The Cancer Imaging Archive (TCIA). Finally, it should also be noted that the parameters used for image acquisition, such as repetition time and echo time, can influence the analysis results to a certain extent [45,46].

The incorporation of advanced MRI techniques, such as diffusion-weighted imaging and perfusion imaging, may further refine the accuracy of molecular predictions. 

While our study focused on the efficacy of radiomics-based machine learning models in predicting the *IDH* mutation status using MRI images, we acknowledge the importance of considering the performance comparison with human experts. Unfortunately, our dataset did not include data on predictions made by human experts during the MRI assessment. Therefore, a direct comparison between the performance of our model and that of human experts was not feasible within the scope of this study. However, exploring this comparison in future research would provide valuable insights into the clinical utility of our model and its potential role alongside human expertise in tumor classification.

## 5. Conclusions

Our study sought to determine which of the commonly used MRI sequences, when coupled with radiomics-based machine learning models, holds the greatest potential for non-invasively predicting the *IDH* mutation status. Our retrospective analysis identified contrast-enhanced T1-weighted MRI images as the optimal MRI sequence for this purpose, yielding impressive performance metrics, with an accuracy of up to 79 percent. The administration of contrast agents significantly contributed to this enhanced predictive power. These findings have the potential to influence clinical practice by guiding neuro-oncologists toward a more precise and efficient approach to glioma characterization, ultimately benefitting patients through improved treatment strategies and outcomes.

## Figures and Tables

**Table 1:** Demographic characteristics of patients included in the study cohort, separately for training data and independent test data as well as for the total data.

	Training Data	Independent Test Data	Total Data
Number of patients	85	21	106
Gender (in %)			
Female	45.09	46.05	45.28
Male	54.91	53.95	54.72
Mean age (in years)	43.52	42.85	43.39
*IDH* status (in %)			
Mutated	67.06	66.67	66.98
Non-mutated (wild-type)	32.94	33.33	33.02

**Table 2:** Most important features and their frequencies of selection during 100 runs. Calculations were performed for the 10-feature models using Lasso regression based on the contrast-enhanced T1-weigthed MRI images.

Level of Importance	Feature Name	Number of Runs Included
1	age_at_diagnosis	99
2	original_shape_Flatness	97
3	original_gldm_LargeDependenceHighGrayLevelEmphasis	86
4	original_shape_LeastAxisLength	78
5	original_firstorder_Kurtosis	61
6	original_glszm_LargeAreaHighGrayLevelEmphasis	55
7	original_glcm_DifferenceAverage	52
8	original_ngtdm_Contrast	43
9	original_shape_Maximum2DDiameterColumn	35
10	original_glszm_ZonePercentage	30
11	original_glrlm_LongRunHighGrayLevelEmphasis	29
12–14	original_glcm_Imc1	25
12–14	original_glszm_LowGrayLevelZoneEmphasis	25
12–14	original_ngtdm_Strength	25
15	original_glcm_ClusterShade	23

**Table 3:** Mean discriminatory power (using 100 runs) in the prediction of *IDH* mutation status in gliomas using Lasso regression based on contrast-enhanced T1-weighted MRI images. The models include 7 and 10 features. Values in brackets indicate 95% confidence intervals. All results were calculated using independent test data based on models with newly determined features for each run, on the one hand, and fixed features according to Table 2, on the other.

	Models with 7 Features		Models with 10 Features	
	Different Features	Fixed Features	Different Features	Fixed Features
AUC	0.743 [0.490:0.933]	0.846 [0.634:0.984]	0.757 [0.511:0.939]	0.842 [0.630:0.984]
Accuracy	0.731 [0.549:0.905]	0.792 [0.596:0.905]	0.743 [0.549:0.905]	0.795 [0.619:0.905]
Sensitivity	0.809 [0.643:1.000]	0.847 [0.643:1.000]	0.824 [0.643:1.000]	0.844 [0.643:1.000]
Specificity	0.576 [0.143:0.857]	0.681 [0.286:1.000]	0.580 [0.218:0.925]	0.696 [0.286:1.000]
PPV	0.798 [0.657:0.929]	0.848 [0.680:1.000]	0.801 [0.667:0.962]	0.855 [0.714:1.000]
NPV	0.618 [0.311:1.000]	0.710 [0.355:1.000]	0.641 [0.333:1.000]	0.716 [0.429:1.000]

## Data Availability

Data available on request. The data presented in this study are available on reasonable request from the corresponding author.
